# Structures of three members of Pfam PF02663 (FmdE) implicated in microbial methanogenesis reveal a conserved α+β core domain and an auxiliary C-terminal treble-clef zinc finger

**DOI:** 10.1107/S1744309110020166

**Published:** 2010-08-04

**Authors:** Herbert L. Axelrod, Debanu Das, Polat Abdubek, Tamara Astakhova, Constantina Bakolitsa, Dennis Carlton, Connie Chen, Hsiu-Ju Chiu, Thomas Clayton, Marc C. Deller, Lian Duan, Kyle Ellrott, Carol L. Farr, Julie Feuerhelm, Joanna C. Grant, Anna Grzechnik, Gye Won Han, Lukasz Jaroszewski, Kevin K. Jin, Heath E. Klock, Mark W. Knuth, Piotr Kozbial, S. Sri Krishna, Abhinav Kumar, Winnie W. Lam, David Marciano, Daniel McMullan, Mitchell D. Miller, Andrew T. Morse, Edward Nigoghossian, Amanda Nopakun, Linda Okach, Christina Puckett, Ron Reyes, Natasha Sefcovic, Henry J. Tien, Christine B. Trame, Henry van den Bedem, Dana Weekes, Tiffany Wooten, Qingping Xu, Keith O. Hodgson, John Wooley, Marc-André Elsliger, Ashley M. Deacon, Adam Godzik, Scott A. Lesley, Ian A. Wilson

**Affiliations:** a Stanford Synchrotron Radiation Lightsource, SLAC National Accelerator Laboratory, Menlo Park, CA, USA; b Joint Center for Structural Genomics, http://www.jcsg.org, USA; c Protein Sciences Department, Genomics Institute of the Novartis Research Foundation, San Diego, CA, USA; d Center for Research in Biological Systems, University of California, San Diego, La Jolla, CA, USA; e Program on Bioinformatics and Systems Biology, Sanford–Burnham Medical Research Institute, La Jolla, CA, USA; f Department of Molecular Biology, The Scripps Research Institute, La Jolla, CA, USA; g Protein Therapeutics Department, Genomics Institute of the Novartis Research Foundation, San Diego, CA, USA; h Photon Science, SLAC National Accelerator Laboratory, Menlo Park, CA, USA

**Keywords:** Pfam family PF02663, metalloproteins, domain swapping, structural genomics, methanogenesis

## Abstract

Examination of the genomic context for members of the FmdE Pfam family (PF02663), such as the protein encoded by the *fmdE* gene from the methanogenic archaeon *Methanobacterium thermoautotrophicum*, indicates that 13 of them are co-transcribed with genes encoding subunits of molybdenum formylmethanofuran dehydrogenase (EC 1.2.99.5), an enzyme that is involved in microbial methane production. Here, the first crystal structures from PF02663 are described, representing two bacterial and one archaeal species: B8FYU2_DESHY from the anaerobic dehalogenating bacterium *Desulfito­bacterium hafniense* DCB-2, Q2LQ23_SYNAS from the syntrophic bacterium *Syntrophus aciditrophicus* SB and Q9HJ63_THEAC from the thermoacidophilic archaeon *Thermoplasma acidophilum*. Two of these proteins, Q9HJ63_THEAC and Q2LQ23_SYNAS, contain two domains: an N-terminal thioredoxin-like α+β core domain (NTD) consisting of a five-stranded, mixed β-sheet flanked by several α-helices and a C-terminal zinc-finger domain (CTD). B8FYU2_DESHY, on the other hand, is composed solely of the NTD. The CTD of Q9HJ63_THEAC and Q2LQ23_SYNAS is best characterized as a treble-clef zinc finger. Two significant structural differences between Q9HJ63_THEAC and Q2LQ23_SYNAS involve their metal binding. First, zinc is bound to the putative active site on the NTD of Q9HJ63_THEAC, but is absent from the NTD of Q2LQ23_SYNAS. Second, whereas the structure of the CTD of Q2LQ23_SYNAS shows four Cys side chains within coordination distance of the Zn atom, the structure of Q9HJ63_THEAC is atypical for a treble-cleft zinc finger in that three Cys side chains and an Asp side chain are within coordination distance of the zinc.

## Abstract 2

The first structures from the FmdE Pfam family (PF02663) reveal that some members of this family form tightly intertwined dimers consisting of two domains (N-terminal α+β core and C-terminal zinc-finger domains), whereas others contain only the core domain. The presence of the zinc-finger domain suggests that some members of this family may perform functions associated with transcriptional regulation, protein–protein interaction, RNA binding or metal-ion sensing.

## 1. Introduction

The Pfam family PF02663 (FmdE; Finn *et al.*, 2008 ▶) currently contains 204 proteins from 74 bacterial and 39 archaeal species (Pfam v.24; http://pfam.sanger.ac.uk/). In thermophilic methanogenic archaea, co-transcription of the *fmdE* gene with downstream genes encoding catalytic subunits of formylmethanofuran dehydrogenase (EC 1.2.99.5) has been reported (Hochheimer *et al.*, 1996 ▶, 1998 ▶; Vorholt *et al.*, 1996 ▶). Formylmethanofuran dehydrogenase is a multi-subunit enzyme that contains tungsten (Bertram *et al.*, 1994 ▶) or molybdenum as well as iron–sulfur clusters (Hochheimer *et al.*, 1996 ▶), and catalyzes the first step in the formation of methane from carbon dioxide in methanogenic and sulfate-reducing microorganisms (Thauer *et al.*, 2008 ▶; Hallam *et al.*, 2004 ▶; Liu & Whitman, 2008 ▶). The proximity of *fmdE* to genes encoding the catalytic subunits suggests a role in methanogenesis for proteins in PF02663. These observations are consistent with environmental genomic studies, in which the *fmdE* gene was identified in microorganisms from anaerobic marine sediments which are believed to have a significant impact on the global environment by consuming methane (reverse methanogenesis), affecting the levels of atmospheric methane as a greenhouse gas (Hallam *et al.*, 2004 ▶).

The genomes of many nonmethanogenic microorganisms also encode proteins in PF02663. Genes from three microbes, *DSY1837* from *Desulfitobacterium hafniense* DCB-2 (UniProt B8FYU2_DESHY), an anaerobic dehalogenating bacterium; *Ta1109* from *Thermoplasma acidophilum* (UniProt Q9HJ63_THEAC), a thermoacidophilic archaeon; and *SYN_00638* from *Syntrophus aciditrophicus* SB (UniProt Q2LQ23_SYNAS), a syntrophic bacterium, encode proteins with molecular weights of 17.4, 23.1 and 21.5 kDa with calculated isoelectric points of 5.95, 6.13 and 6.21, respectively. Their structures, which are the first reported for the PF02663 Pfam family, were determined using the semi-automated high-throughput pipeline of the Joint Center for Structural Genomics (JCSG; Lesley *et al.*, 2002 ▶) as part of the NIH National Institute of General Medical Sciences’ Protein Structure Initiative (PSI).

## 2. Materials and methods

### 2.1. Protein production and crystallization

Clones for *DSY1837*, *Ta1109* and *SYN_00638* were generated using the Polymerase Incomplete Primer Extension (PIPE) cloning method (Klock *et al.*, 2008 ▶). The gene encoding DSY1837 (GenBank YP_002459451.1; UniProt B8FYU2_DESHY) was amplified by polymerase chain reaction (PCR) from *D. hafniense* DCB-2 genomic DNA using *PfuTurbo* DNA polymerase (Stratagene) and I-PIPE primers (forward, 5′-ctgtacttccagggcATGTGCGTAGAAAAAACC­CCTTGGGAAC-3′; reverse, 5′-aattaagtcgcgttaAACTATTTTACTC­AGTTGTCCCGGA-3′; target sequence in upper case) that included sequences for the predicted 5′ and 3′ ends. The expression vector pSpeedET, which encodes an amino-terminal tobacco etch virus (TEV) protease-cleavable expression and purification tag (MGSDK­IHHHHHHENLYFQ/G), was PCR-amplified with V-PIPE (Vector) primers. V-PIPE and I-PIPE PCR products were mixed to anneal the amplified DNA fragments together. *Escherichia coli* GeneHogs (Invitrogen) competent cells were transformed with the V-PIPE/I-­PIPE mixture and dispensed onto selective LB–agar plates. The cloning junctions were confirmed by DNA sequencing. Expression was performed in a selenomethionine-containing medium at 310 K. Cells were induced after 1.5 h using 0.11%(*w*/*v*) arabinose and were allowed to grow for an additional 3 h before harvesting. Selenomethionine was incorporated *via* inhibition of methionine biosynthesis (Van Duyne *et al.*, 1993 ▶), which does not require a methionine-auxotrophic strain.

At the end of fermentation, lysozyme was added to the culture to a final concentration of 250 µg ml^−1^ and the cells were harvested and frozen. After one freeze–thaw cycle, the cells were sonicated in lysis buffer [50 m*M* HEPES pH 8.0, 50 m*M* NaCl, 10 m*M* imidazole, 1 m*M* tris(2-carboxyethyl)phosphine–HCl (TCEP)] and the lysate was clarified by centrifugation at 32 500*g* for 30 min. The soluble fraction was passed over nickel-chelating resin (GE Healthcare) pre-equilibrated with lysis buffer, the resin was washed with wash buffer [50 m*M* HEPES pH 8.0, 300 m*M* NaCl, 40 m*M* imidazole, 10%(*v*/*v*) glycerol, 1 m*M* TCEP] and the protein was eluted with elution buffer [20 m*M* HEPES pH 8.0, 300 m*M* imidazole, 10%(*v*/*v*) glycerol, 1 m*M* TCEP]. The eluate was buffer-exchanged with TEV buffer (20 m*M* HEPES pH 8.0, 200 m*M* NaCl, 40 m*M* imidazole, 1 m*M* TCEP) using a PD-10 column (GE Healthcare) and incubated with 1 mg TEV protease per 15 mg of eluted protein. The protease-treated eluate was run over nickel-chelating resin (GE Healthcare) pre-equilibrated with HEPES crystallization buffer (20 m*M* HEPES pH 8.0, 200 m*M* NaCl, 40 m*M* imidazole, 1 m*M* TCEP) and the resin was washed with the same buffer. The flowthrough and wash fractions were combined and concentrated to 15 mg ml^−1^ by centrifugal ultrafiltration (Millipore) for crystallization trials. B8FYU2_DESHY was crystallized at 277 K using the nanodroplet vapor-diffusion method (Santarsiero *et al.*, 2002 ▶) with standard JCSG crystallization protocols (Lesley *et al.*, 2002 ▶). The crystallization reagent used was composed of 0.2 *M* MgCl_2_ and 20.0% PEG 3350. Ethylene glycol was added to the crystal as a cryoprotectant to a final concentration of 10%(*v*/*v*). Initial screening for diffraction was carried out using the Stanford Automated Mounting system (SAM; Cohen *et al.*, 2002 ▶) at the Stanford Synchrotron Radiation Lightsource (SSRL, Menlo Park, California, USA). The crystal was indexed in the primitive orthorhombic space group *P*2_1_2_1_2_1_. The oligomeric state of B8FYU2_DESHY in solution was determined using a 1 × 30 cm Superdex 200 size-exclusion column (GE Healthcare; Klock *et al.*, 2008 ▶) coupled with miniDAWN (Wyatt Technology) static light-scattering (SEC/SLS) and Optilab differential refractive-index detectors (Wyatt Technology). The mobile phase consisted of 20 m*M* Tris pH 8.0, 150 m*M* NaCl and 0.02%(*w*/*v*) sodium azide.

The *Ta1109* gene (GenBank CAC12236.1; UniProt ID Q9HJ63_THEAC) was amplified from *T. acidophilum* DSM1728 genomic DNA. Cloning (forward primer, 5′-ctgtacttccagggcATGGAGAAA­CTGAATTTCGGAATTCCAG-3′; reverse primer, 5′-aattaagtcgcgt­taTTTCTTGCCGTAGTAATCAGGCTTGCAC-3′; target sequence in upper case), expression and purification were performed as described for B8FYU2_DESHY. Purified Q9HJ63_THEAC was concentrated to 14 mg ml^−1^ for crystallization trials and was crystallized at 277 K using the nanodroplet vapor-diffusion method (Santarsiero *et al.*, 2002 ▶) with standard JCSG crystallization protocols (Lesley *et al.*, 2002 ▶). The crystallization reagent used was composed of 0.2 *M* magnesium nitrate and 20.0% PEG 3350. The crystal was indexed in the monoclinic space group *C*2. A second crystal was obtained using a solution consisting of 10.0% PEG 8000, 0.2 *M* zinc acetate and 0.1 *M* MES pH 6.0. These crystals were indexed in the *I*-­centered orthorhombic space group *I*222. A third crystal was grown in a solution consisting of 0.2 *M* magnesium nitrate and 20.0% PEG 3350 and was indexed in the tetragonal space group *P*4_2_2_1_2. Ethylene glycol was added to the crystals as cryoprotectant to a final concentration of 15%(*v*/*v*). Initial screening for diffraction and oligomeric state determination were performed as described for B8FYU2_DESHY.

The *SYN_00638* gene (GenBank CP000252; UniProt Q2LQ23_SYNAS) was amplified from *S. aciditrophicus* SB genomic DNA. Cloning (forward primer, 5′-ctgtacttccagggcATGACAGCACGTAA­TATTTTGTCTTAC-3′; reverse primer, 5′-aattaagtcgcgttaAAGAT­AAGGCGACCCTCCCTGGCAGCTC-3′; target sequence in upper case), expression and purification were performed as described for B8FYU2_DESHY. Purified Q2LQ23_SYNAS was concentrated to 20 mg ml^−1^ for crystallization trials and was crystallized at 277 K using the nanodroplet vapor-diffusion method (Santarsiero *et al.*, 2002 ▶) with standard JCSG crystallization protocols (Lesley *et al.*, 2002 ▶). The crystallization reagent was composed of 0.01 *M* nickel chloride, 20.0% PEG MME 2000 and 0.1 *M* Tris pH 8.5. Glycerol was added to the crystal as a cryoprotectant to a final concentration of 10%(*v*/*v*). Initial screening for diffraction and oligomeric state determination were carried out as described for B8FYU2_DESHY. The crystal was indexed in the tetragonal space group *P*4_1_2_1_2.

### 2.2. Data collection, structure solution and refinement

X-ray diffraction data were collected on beamline 9-2 at the Stanford Synchrotron Radiation Lightsource (SSRL) at wavelengths corresponding to the high-energy remote (λ_1_), inflection (λ_2_) and peak (λ_3_) wavelengths of a three-wavelength selenium multi-wavelength anomalous diffraction (Se-MAD) experiment for the *P*2_1_2_1_2_1_ crystal form of B8FYU2_DESHY and the *C*2 crystal form of Q9HJ63_THEAC. Three-wavelength Se-MAD data were collected on beamline 11-1 at SSRL for Q2LQ23_SYNAS. Additional diffraction data for Q9HJ63_THEAC were collected from the two other crystal forms (*I*222 and *P*4_2_2_1_2) on beamlines 11-1 and 9-2 at SSRL at wavelengths of 1.00 and 0.9790 Å, respectively. MAD phasing for Q9HJ63_THEAC was carried out using the *C*2 crystal data and further refinement was performed using the *I*222 data at a higher resolution of 1.87 Å after molecular replacement with *Phaser* (McCoy, 2007 ▶) using the model obtained from the *C*2 data. All data sets were collected at 100 K using either an ADSC Quantum 315 detector (beamline 11-1) or a MAR Mosaic 325 CCD detector (beamline 9-2). The data were integrated and scaled using either *MOSFLM* (Leslie, 1992 ▶) and *SCALA* from the *CCP*4 program suite (Collaborative Computational Project, Number 4, 1994 ▶) or the *XDS* and *XSCALE* programs (Kabsch, 1993 ▶, 2010*a* ▶,*b* ▶). Data statistics are summarized in Table 1 ▶ for B8FYU2_DESHY, in Tables 2 ▶ and 3 ▶ for Q9HJ63_THEAC and in Table 4 ▶ for Q2LQ23_SYNAS. The selenium substructures for the three proteins were solved with *SHELXD* (Sheldrick, 2008 ▶) and the MAD phases were refined with *autoSHARP* for Q9HJ63_THEAC and Q2LQ23_SYNAS (Vonrhein *et al.*, 2007 ▶) and *SOLVE* (Terwilliger & Berendzen, 1999 ▶) for B8FYU2_DESHY. The mean figures of merit were 0.45, 0.37 and 0.35, respectively. Automatic model building was performed with either *ARP*/*wARP* (Cohen *et al.*, 2004 ▶) or *RESOLVE* (Terwilliger, 2002 ▶). Model completion was performed using *Coot* (Emsley & Cowtan, 2004 ▶) and refinement was accomplished using *REFMAC*5 (Winn *et al.*, 2003 ▶). Refinement statistics are summarized in Tables 1 ▶, 3 ▶ and 4 ▶ for B8FYU2_DESHY, Q9HJ63_THEAC and Q2LQ23_SYNAS, respectively.

### 2.3. Validation and deposition

**Figure 1:**
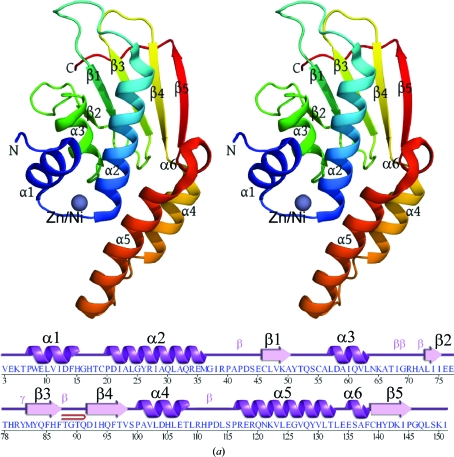

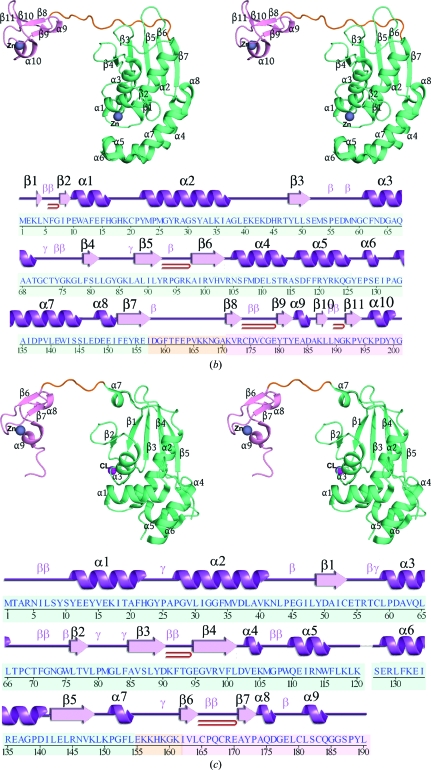
Crystal structures of (*a*) B8FYU2_DESHY, (*b*) Q9HJ63_THEAC and (*c*) Q2LQ23_SYNAS. The polypeptide backbones are shown as stereo ribbon diagrams. Below the ribbon representations are the secondary-structure elements superimposed on the primary sequence. The α-helices, 3_10_-helices, β-strands, β-turns and γ-turns are indicated. β-Hairpins are depicted as red loops. (*a*) For B8FYU2_DESHY, the protein ribbon is color-coded from the N-terminus (blue) to the C-terminus (red). Helices α1–α4 and β-­strands (β1–β6) are indicated. A dual-occupancy zinc/nickel-binding site in the vicinity of the putative active site on the α+β core and the zinc-finger domain is shown as a gray sphere. (*b*) For Q9HJ63_THEAC, helices α1–α10 and β-strands (β1–β11) are indicated. The subregions of the structure, the core domain (NTD), linker and C-terminal zinc-finger domain (CTD), and the background of the corresponding sequence are colored turquoise, orange and pink, respectively. Zn atoms are shown as gray spheres. (*c*) For Q2LQ23_SYNAS, helices H1–H10 and β-strands (β1–β7) are indicated with subregions of the structure colored as in (*b*). A chloride ion in the vicinity of the putative active site is shown as a magenta sphere and the Zn atom bound to the zinc-finger domain is shown as a gray sphere.

The quality of the crystal structure was analyzed using the *JCSG Quality Control* server (see http://smb.slac.stanford.edu/jcsg/QC/). This server verifies the stereochemical quality of the model using *AutoDepInputTool* (Yang *et al.*, 2004 ▶), *MolProbity* (Chen *et al.*, 2010 ▶) and *WHAT IF* v.5.0 (Vriend, 1990 ▶), the agreement between the atomic model and the data using *SFCHECK* v.4.0 (Vaguine *et al.*, 1999 ▶) and *RESOLVE* (Terwilliger, 2002 ▶), the protein sequence using *ClustalW* (Chenna *et al.*, 2003 ▶), the atom occupancies using *MOLEMAN*2 (Kleywegt *et al.*, 2001 ▶) and the consistency of NCS pairs. It also evaluates differences in *R*_cryst_/*R*_free_, expected *R*_free_/*R*_cryst_ and maximum/minimum *B* values by parsing the refinement log file and PDB header. The EBI *PISA* server (Krissinel & Henrick, 2007 ▶) was used to analyze the protein quaternary structure. Figs. 1(*a*), 1(*b*) and 1(*c*) were adapted from *PDBsum* (Laskowski, 2009 ▶) and the other figures were prepared using *PyMOL* (DeLano Scientific). Atomic coordinates and experimental structure factors for B8FYU2_DESHY at 1.45 Å resolution, Q9HJ63_THEAC at 1.87 Å resolution and Q2LQ23_SYNAS at 1.90 Å resolution have been deposited in the PDB and are accessible under codes 2glz, 2gvi and 3d00, respectively.

## 3. Results and discussion

### 3.1. Overall structures

The crystal structure of B8FYU2_DESHY (Fig. 1 ▶ *a*) was determined by MAD at 1.45 Å resolution. Data-collection, model and refinement statistics are summarized in Table 1 ▶. The final model includes two protein molecules (residues 3–151 for chain *A*; residues 4–151 for chain *B*), 18 ethylene glycol molecules, one Zn atom, one Ni atom and 427 water molecules in the asymmetric unit. No electron density was observed for a few residues at the N- and C-termini of both chains (Gly*A*0, Mse*A*1, Cys*A*2, Val*A*152, Gly*B*0, Mse*B*1, Cys*B*2, Val*B*3 and Val*B*152) or for side-chain atoms of Val*A*3, Glu*A*4, Asp*A*43, Arg*A*117, Glu*A*118, Arg*A*119, Ile*A*151, Glu*B*4, Asp*B*43, His*B*111, Asp*B*113, Arg*B*117 and Ile*B*151. The Matthews coefficient (*V*_M_; Matthews, 1968 ▶) was 2.82 Å^3^ Da^−1^ and the estimated solvent content was 56.4%. The Ramachandran plot produced by *MolProbity* (Davis *et al.*, 2004 ▶) showed that 99% of the residues are in favored regions, with no outliers. B8FYU2_DESHY is composed of five β-­strands (β1–β5) and six α-helices (α1–α6) (Fig. 1 ▶ *a*). The total β-­sheet and α-helical contents are 24% and 58%, respectively. The monomer consists of a central five-stranded, mixed β-sheet (21345 topology) with one solvent-exposed face, while the other is covered by three α-­helices. A distinctive feature of the structure is the protrusion of two helices (α4 and α5) and a connecting loop (residues 99–138) from the core of each molecule.

The crystal structure of Q9HJ63_THEAC (Fig. 1 ▶ *b*) was initially determined by MAD from the *C*2 crystal form at 2.0 Å resolution. Molecular replacement was then used to determine the structure of the *I*222 crystal form at 1.87 Å resolution. Data-collection, model and refinement statistics are summarized in Tables 2 ▶ and 3 ▶. The final model includes one protein molecule (residues 1–201), one unknown ligand (UNL), five Zn atoms, six ethylene glycol molecules, eight acetate ions and 129 water molecules in the asymmetric unit. No electron density was observed for a few residues at the N- and C-­termini (Gly0, Lys202 and Lys203) or for side-chain atoms of Mse1, Glu2, Lys3, Arg117, Glu10, Lys35, Arg155, Glu163 and Lys192. The Matthews coefficient (*V*_M_; Matthews, 1968 ▶) for the *I*222 form was 2.87 Å^3^ Da^−1^ and the estimated solvent content was 56.8%. The Ramachandran plot produced by *MolProbity* (Davis *et al.*, 2004 ▶) showed that 99% of the residues are in favored regions, with no outliers. Q9HJ63_THEAC is composed of 11 β-strands (β1–β11) and ten α-helices (α1–α10) (Fig. 1 ▶ *b*). The total β-sheet, α-helical and 3_10_-­helical contents are 24, 58 and 2.5%, respectively. In addition to the N-terminal α+β core domain (NTD; residues 1–157), which is similar to that of B8FYU2_DESHY, Q9HJ63_THEAC also has a C-­terminal domain (CTD) with a treble-clef, zinc finger-like motif (Grishin, 2001 ▶; residues 169–201); it is connected to the N-­terminal domain *via* an 11-residue linker.

The crystal structure of Q2LQ23_SYNAS (Fig. 1 ▶ *c*) was determined by MAD at 1.90 Å resolution. Data-collection, model and refinement statistics are summarized in Table 4 ▶. The final model includes one protein molecule (residues 1–190), one chloride anion, one Zn atom and 42 water molecules in the asymmetric unit. The smaller than expected number of ordered water molecules for a 1.9 Å resolution structure coincides with elevated *R*_cryst_ and *R*_free_ values of 23.3% and 26.8%, respectively. One possible explanation for the larger than expected *R* values is the anisotropy of the diffraction intensities, with a spread in the values of the three principal components of 21.4 Å^2^ and with diffraction intensity falling off more significantly in the a* and b* directions compared with the c* direction. No electron density was observed for residues *A*121–*A*126 or for side-chain atoms of Glu*A*16, Lys*A*17, Asp*A*48, Arg*A*56, Glu*A*95, Lys*A*105, Gln*A*110, Lys*A*118, Lys*A*120, Glu*A*128, Arg*A*129, Lys*A*132, Glu*A*136, Lys*A*148, Lys*A*150, Glu*A*155, Lys*A*156, Lys*A*157, His*A*158, Lys*A*159 and Lys*A*161. The Matthews coefficient (*V*_M_; Matthews, 1968 ▶) for Q2LQ23_SYNAS was 2.33 Å^3^ Da^−1^ and the estimated solvent content was 47.1%. The Ramachandran plot produced by *MolProbity* showed that 96.1% of the residues are in favored regions, with no outliers. Q2LQ23_SYNAS (Fig. 1 ▶ *c*) is composed of seven β-­strands (β1–β7) and nine α-helices (α1–α9). The total β-sheet, α-helical and 3_10_-helical contents are 18, 56 and 4.9%, respectively. Q2LQ23_SYNAS displays a similar architecture to Q2HJ63_THEAC, with a larger NTD (residues 1–154) and a smaller, treble-clef zinc-finger domain CTD coupled together through a nine-residue linker (residues 155–163). The linkers in Q9HJ63_THEAC and Q2LQ23_SYNAS separate the NTD and CTD domains so that the closest edges of the two domains are ∼20 Å apart.

### 3.2. Oligomerization

**Figure 2:**
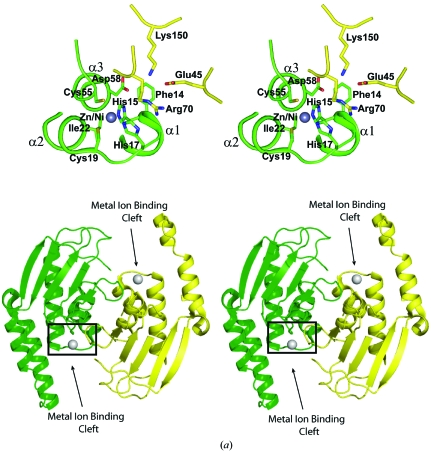

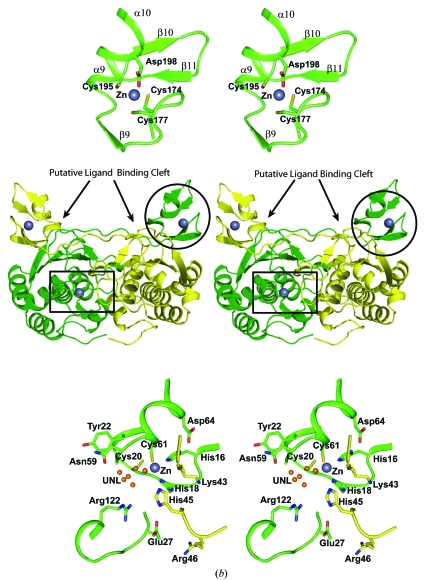

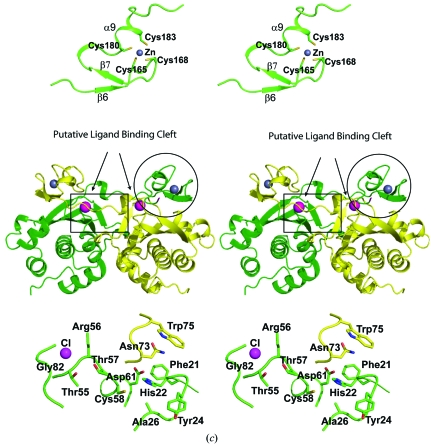
Stereo ribbon representations and close-up views of the structure surrounding the metal ion-binding sites in (*a*) B8FYU2_DESHY, (*b*) Q9HJ63_THEAC and (*c*) Q2LQ23_SYNAS. (*a*) Stereo diagram of the structure surrounding one of the zinc/nickel-binding sites (top) of the B8FYU2_DESHY dimer (bottom) and indicated by a rectangle. The metal ion-binding clefts on the dimer are indicated. (*b*) Stereo diagram of one of the zinc-binding sites on the α+β core domains (bottom), on the Q9HJ63_THEAC dimer (middle) and on one of the zinc-finger domains (top). The sites on the NTD and CTD are indicated by a rectangle and a circle, respectively. An unidentified ligand (UNL) modeled at the putative active site on the α+β core domain in the *I*222 crystal form is shown as orange spheres. A large putative binding cleft on the surface of the dimer is indicated. (*c*) Stereo diagram of one of the putative active-site clefts (bottom; indicated by a rectangle), the Q2LQ23_SYNAS dimer (middle) and one of the zinc-finger domains (top; indicated by a circle). The O, N, and S atoms on the side chains are shown in red, blue and yellow, respectively. Bound metal atoms and chloride anions are shown as gray and magenta spheres, respectively. A large putative binding cleft on the surface of the dimer is indicated.

B8FYU2_DESHY, Q9HJ63_THEAC and Q2LQ23_SYNAS contain stable dimeric interfaces of 2030, 5860 and 4350 Å^2^, respectively, as predicted by *PISA* (Krissinel & Henrick, 2007 ▶). Analytical size-exclusion chromatography coupled with static light scattering also supports these assignments in solution, suggesting that a dimer is the functionally relevant oligomer for each. The asymmetric unit dimer for B8FYU2_DESHY is approximately S-shaped, with several close-range monomer–monomer interactions between residues on helix α3 (Fig. 2 ▶ *a*). The dimer has two prominent C-shaped grooves that extend along its surface parallel to the twofold axis; they are ∼15 Å wide and are exposed to solvent at either end (Fig. 2 ▶ *a*). All crystal forms of Q9HJ63_THEAC (Fig. 2 ▶ *b*) and Q2LQ23_SYNAS (Fig. 2 ▶ *c*) show similar twofold-symmetric, domain-swapped dimers in which the NTD and the CTD of one polypeptide chain are separated by an 11-­residue linker and the CTD is anchored to the NTD of the symmetry-related monomer. Analysis of the structures of the Q9HJ63_THEAC and Q2LQ23_SYNAS dimers using *CASTp* (Dundas *et al.*, 2006 ▶) shows an ∼20 Å wide surface depression (Figs. 2 ▶ *b* and 2 ▶ *c*) that is large enough to accommodate a fairly large ligand.

### 3.3. Metal-ion binding in the NTD

A metal ion-binding site was identified at the bottom of the C-­shaped groove in B8FYU2_DESHY (Fig. 2 ▶ *a*). The metal ion is solvent-accessible and within coordination distance of His15, His17, Cys19 and Cys55 (Fig. 2 ▶ *a*, Table 5 ▶). X-ray anomalous scattering measurements indicated that the site had a mixed occupancy of zinc and nickel. The total occupancy of the zinc and nickel cations was reduced to 0.75 to match the observed scattering at this site, with a zinc:nickel ion stoichiometric ratio of 2.6:1 estimated from the ratio of their anomalous difference map peak heights. The guanidinium side chain of Arg70 from the other subunit in the dimer is within hydrogen-bonding distance of the carbonyl O atom of His15 and stacks parallel to the side chain of His17, which coordinates the metal (Fig. 2 ▶ *a*).

X-ray fluorescence emission spectroscopy from the *C*2 crystals of Q9HJ63_THEAC indicated the presence of zinc. To corroborate that zinc was bound at specific sites in the structure and not just in the bulk solvent, anomalous difference maps were calculated from data collected at wavelengths above and below the zinc X-ray absorption edge. One of the binding sites was located on the NTD (Fig. 2 ▶ *b*, Table 5 ▶) and a second on the CTD (Fig. 2 ▶ *b*, Table 5 ▶). All three crystal forms show zinc binding at the same two sites, suggesting that these sites are functionally relevant (note that two of the three crystal forms, *C*2 and *P*4_2_2_1_2, are devoid of exogenous zinc in the crystallization conditions). The *I*222 crystal form also showed four additional zinc-binding sites, which are likely to be attributable to the presence of zinc acetate in the crystallization experiments.

In Q9HJ63_THEAC the zinc-binding site on the NTD is situated on a loop connecting the N-terminal α-helices (α1 and α2). The zinc is within coordination distance of His16, His18, Cys20 and Cys61 (Fig. 2 ▶ *b*). These side chains are conserved in B8FYU2_DESHY, in which the NTD metal ion-binding site occupies a similar position. In the *I*222 crystal form of Q9HJ63_THEAC, unexplained electron density near the zinc and Cys61 was modeled as an unknown ligand (UNL; Fig. 2 ▶ *b*). The UNL is only 1.8 Å from the S atom of the conserved Cys61, which is consistent with a thioester bond between the protein and the UNL.

This binding site and the UNL are located within an elongated cleft on the surface of the dimer that is approximately 30 Å long and 10 Å wide (Fig. 2 ▶ *b*). Each dimer contains two symmetry-related clefts positioned ∼25 Å apart that are assembled from both subunits, including portions of the zinc-finger domain and its β-strand bridging the N- and C-terminal domains. In Q2LQ23_SYNAS no zinc is bound to the NTD. It is worth noting that two of the zinc-binding residues in B8FYU2_DESHY and Q9HJ63_THEAC are not conserved in Q2LQ23_SYNAS: His15 and Cys19 (B8FYU2_DESHY numbering) are replaced by Tyr and Ala, respectively (Fig. 2 ▶ *c*). Instead, an occupied anion-binding site was identified in Q2LQ23_SYNAS (Fig. 2 ▶ *c*) and was modeled as a chloride based on the electron density being within 3.5 Å of the polypeptide backbone N atoms of Arg56 and Gly82 and the presence of chloride in the crystallization reagent. The chloride is bound near the end of the central β-sheet facing towards the extended stretch of polypeptide connecting the NTD and the CTD on the symmetry-related subunit.

### 3.4. Metal-ion binding in the CTD

The bound zinc on the zinc-finger domain of Q9HJ63_THEAC shows a somewhat atypical coordination mode, with the side chains of Cys174, Cys177, Cys195 and Asp198 within ligation distance (Fig. 2 ▶ *b*, Table 5 ▶). Typically, zinc ions in treble-clef zinc fingers are within co­ordination distance of Cys or His residues. Atypical coordination modes in which Asp or Glu act as ligands for the zinc have been observed previously in the zinc-finger domains of the mouse LIM–ldb1 LID complex (Deane *et al.*, 2004 ▶; PDB code 1rut), the human integrin-linked kinase ankyrin-repeat domain in complex with the PINCH1 LIM1 domain (Chiswell *et al.*, 2008 ▶; PDB code 3f6q), LIM domains 1 and 2 in complex with the LIM-interacting domain of LDB1 from mouse (Jeffries *et al.*, 2006 ▶; PDB code 2dfy) and the heterodimeric core primase from *Sulfolobus solfataricus* (Lao-Sirieix *et al.*, 2005 ▶; PDB code 1zt2). Recently, the structure of a prokaryotic homolog of the transcriptional regulator of Ros from *Agrobacterium tumefaciens* was reported in which an Asp also replaces a Cys as a zinc ligand in the Cys_2_His_2_ domain (Baglivo *et al.*, 2009 ▶). Q2LQ23_SYNAS also has a single zinc-binding site on the zinc-finger domain, although here the zinc-chelating residues (Cys165, Cys168, Cys180 and Cys183; Fig. 2 ▶ *c*, Table 5 ▶) are more typical.

### 3.5. Structural comparisons of the PF02663 proteins

Whereas 48 PF02663 proteins, including B8FYU2_DESHY, are comprised of only a single NTD-like sequence motif, 98 others, including Q9HJ63_THEAC and Q2LQ23_SYNAS, also contain a C-­terminal extension of ∼40 amino acids with conserved cysteine and aspartic acid residues. The structures of Q9HJ63_THEAC and Q2LQ23_SYNAS show that these conserved residues form a zinc-binding site on a zinc-finger domain. Two other proposed domain architectures in the PF02263 family, for which structures have not yet been determined, include an NTD fused to a molybdopterin-binding domain (PF00994) and an NTD fused to a domain from the un­characterized protein family UPF0066 (PF01980).

**Figure 3:**
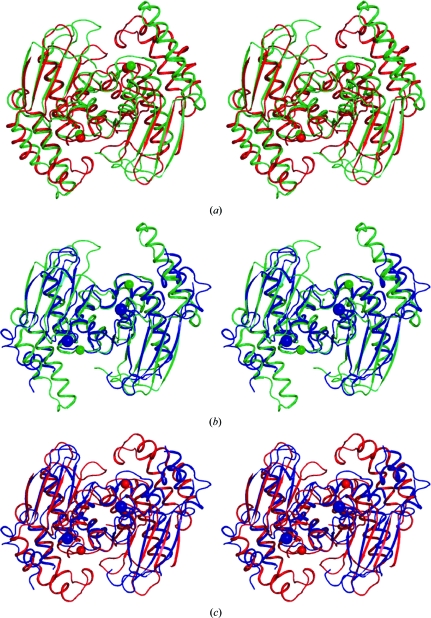
Pairwise comparison of the α+β core-domain structures of three PF02663 homologs. (*a*) Stereo diagram showing the superposition of the ribbon traces for (*a*) B8FYU2_DESHY (PDB code 2glz; green) and Q9HJ63_THEAC (PDB code 2gvi; red). The Zn/Ni atoms in B8FYU2_DESHY are shown as green spheres and the Zn atoms from Q9HJ63_THEAC are shown as red spheres. (*b*) Stereo diagram showing the superposition of the ribbon traces for B8FYU2_DESHY (PDB code 2glz; green) and Q2LQ23_SYNAS (PDB code 3d00; blue). The Zn/Ni atoms in B8FYU2_DESHY are shown as green spheres and the chloride ions from Q2LQ23_SYNAS are shown as blue spheres. (*c*) Stereo diagram showing the superposition of the ribbon traces for Q9HJ63_THEAC (PDB code 2gvi; red) and Q2LQ23_SYNAS (PDB code 3d00; blue). The Zn/Ni atoms in Q9HJ63_THEAC are shown as red spheres and the chloride ions from Q2LQ23_SYNAS are shown as blue spheres.

Pairwise structural comparisons of B8FYU2_DESHY, Q9HJ63_THEAC and Q2LQ23_SYNAS (Fig. 3 ▶) revealed that the NTDs of B8FYU2_DESHY and Q9HJ63_THEAC are the most similar. The NTDs of B8FYU2_DESHY and Q9HJ63_THEAC (Fig. 3 ▶ *a*) contain two conserved sequence motifs. The first motif, with a consensus sequence FHGH*x*C (Phe14–Cys19; B8FYU2_DESHY numbering), contains three residues that coordinate the bound metal and is located on a loop connecting α1 and α2 (Figs. 1 ▶ *a* and 1 ▶ *b*). The second motif contains Asp58, Gln61 and Thr67 (B8FYU2_DESHY num­bering) and is located along the twofold-symmetry axis at the dimer interface.

The overall fold of the zinc-finger domains of Q9HJ63_THEAC (residues 171–201) and Q2LQ23_SYNAS (residues 162–190) are similar, with an r.m.s.d. of 1.1 Å for 24 superposed C^α^ atoms. Two conserved Cys residues on the first β-loop of the CTD coordinate zinc (*i.e.* the zinc knuckle). These loops are located between β8 and β9 (Fig. 2 ▶ *b*) and between β6 and β7 (Fig. 2 ▶ *c*) in Q9HJ63_THEAC and Q2LQ23_SYNAS, respectively. The remaining zinc ligands (*i.e.* the two other Cys residues in Q2LQ23_SYNAS and a Cys and an Asp in Q9HJ63_THEAC) are located near the C-terminal α-helix H10 (Figs. 2 ▶ *b* and 2 ▶ *c*).

### 3.6. Comparison with other structures

A *DALI* (Holm & Sander, 1995 ▶) search revealed that the NTD domain of Q9HJ63_THEAC shows structural similarity to the intervening domain of 3-phosphoglycerate dehydrogenase from *Mycobacterium tuberculosis* (PDB code 3dc2; *DALI Z* score = 6.5, 7% sequence identity, 3.1 Å r.m.s.d. overlap of 96 C^α^ atoms; Dey *et al.*, 2008 ▶) and to a fragment from an iron–sulfur-dependent l-serine dehydratase from *Legionella pneumophila* (PDB code 2iqq; *DALI Z* score = 4.3, 7% sequence identity, 2.7 Å r.m.s.d. overlap of 78 C^α^ atoms). The low sequence identity between the NTD and the *DALI* hits suggests alternate functions for PF02663. In addition, four of the five strands in the β-sheet (β1, β2, β3 and β4 in Fig. 1 ▶ *a*) and one of the α-helices (α3 in Fig. 1*a* ▶) on the NTD are topologically equivalent to corresponding secondary-structure elements in the thioredoxin-like fold (Qi & Grishin, 2005 ▶; Martin, 1995 ▶). Therefore, the NTD can be classified as a type I circular permutation of the thioredoxin-like fold (Qi & Grishin, 2005 ▶), although thioredoxins are not reported to contain an equivalent metal ion-binding site, in contrast to the circularly permutated PF02263 NTD.

A *FATCAT* search of the PDB shows that the structure of the zinc-finger CTD on Q9HJ63_THEAC is similar to the individual treble-clef zinc-finger subdomains of several eukaryotic LIM-like proteins (Gamsjaeger *et al.*, 2007 ▶; Krishna *et al.*, 2003 ▶). A similar search shows that the zinc-finger domain of Q2LQ23_SYNAS is structurally similar to the phosphatidylinositol-3-phosphate-specific membrane-targeting binding FYVE domain of vps27p from *Saccharomyces cerevisiae* (Misra & Hurley, 1999 ▶; PDB code 1vfy).

### 3.7. Functional implications

The identification of a treble-clef, zinc-finger domain on Q9HJ63_THEAC and Q2LQ23_SYNAS indicates that some PF02663 family members may be involved in transcriptional regulation or protein–protein interactions. However, since the range of functions per­formed by zinc fingers is diverse, a more detailed functional annotation remains a challenge at present. It has been suggested that a PF02663 homolog in *Methanoscarina barkeri* could be a chaperone (Vorholt *et al.*, 1996 ▶). Chaperone activity has also been proposed based on the structure of thioredoxin-2 from the photosynthetic bacterium *Rhodobacter capsulatus* (Ye *et al.*, 2007 ▶; PDB code 2ppt). However, in contrast to the structures of the three PF02663 proteins described here, the zinc-finger domain is at the N-­terminal end of the protein and the motif for the zinc finger in thioredoxin-2 is a zinc ribbon distinct from the treble-clef motif in the PF02663 structures.

Previous investigations have established that in some organisms *fmdE* is co-transcribed with genes encoding the catalytic subunits of a key methanogenic enzyme. Genome-context analysis indicates that only a handful (13 of 208) of genes corresponding to PF02663 members are adjacent to and likely to be co-transcribed with genes encoding the catalytic subunits of molybdemum formylmethanofuran dehydrogenase. Sequence analyses, combined with the structure determinations described here, indicate that 12 of these genes are likely to be part of an *fmd* operon with a two-domain NTD + zinc-finger architecture, whereas an *fmdE* homolog from *M. barkeri* has a one-domain NTD-like architecture. However, most of the genes encoding PF02663 homologs, irrespective of domain architecture, are adjacent to genes encoding metal-ion transporters. These results indicate the absence of a strict correlation between domain architecture and gene context; nevertheless, the results do suggest a possible involvement in metal-ion transport.

## 4. Conclusions

The structures of three members of PF02663 enhance our understanding of the role of these proteins in microbes. Individual proteins within this family display differences in domain architectures, metal-ion binding propensities and dimer interactions. These structural differences suggest a broad range of potential functions for this group of proteins. The identification of a C-terminal zinc-finger domain in two of the structures suggests one possible role for this class of proteins as transcriptional regulators. The NTD together with the CTD might serve as part of the nucleic acid binding surface and/or serve as a signal-sensing domain for the binding of unknown effectors. The absence of a zinc-finger domain in some PF02663 homologs, such as B8FYU2_DESHY, provides some evidence for involvement in alternate processes. Further biochemical and biophysical studies should yield valuable insights into the relationship between structure and function for this interesting group of proteins.

Additional information about the proteins described in this study is available from TOPSAN (Krishna *et al.*, 2010 ▶) at http://www.topsan.org/explore?PDBid=2glz for B8FYU2_DESHY, http://www.topsan.org/explore?PDBid=2gvi for Q9HJ63_THEAC and http://www.topsan.org/explore?PDBid=3d00 for Q2LQ23_SYNAS.

## Supplementary Material

PDB reference: B8FYU2_DESHY, 2glz

PDB reference: Q9HJ63_THEAC, 2gvi

PDB reference: Q2LQ23_SYNAS, 3d00

## Figures and Tables

**Table 1:** Summary of crystal parameters, data-collection and refinement statistics for B8FYU2_DESHY (PDB entry 2glz) Values in parentheses are for the highest resolution shell.

	λ_1_ MADSe	λ_2_ MADSe	λ_3_ MADSe
Space group	*P*2_1_2_1_2_1_		
Unit-cell parameters (Å)	*a* = 46.42, *b* = 84.79, *c* = 100.71		
Data collection			
Wavelength (Å)	0.91837	0.97927	0.97905
Resolution range (Å)	28.26–1.45 (1.49–1.45)	28.25–1.49 (1.53–1.49)	28.27–1.49 (1.53–1.49)
No. of observations	524343	482691	501396
No. of unique reflections	71199	65618	65729
Completeness (%)	99.9 (99.9)	99.9 (99.5)	99.9 (99.7)
Mean *I*/σ(*I*)	17.9 (1.6)	18.0 (2.1)	18.2 (2.1)
*R*_merge_ on *I*† (%)	7.1 (73.0)	7.6 (58.1)	7.4 (79.8)
*R*_meas_ on *I*‡ (%)	7.6 (82.3)	8.1 (65.6)	7.9 (85.6)
Model and refinement statistics			
Resolution range (Å)	27.2–1.45		
No. of reflections (total)	71126		
No. of reflections (test)	3593		
Completeness (%)	99.8		
Data set used in refinement	λ_1_ MADSe		
Cutoff criterion	|*F*| > 0		
*R*_cryst_§	0.171		
*R*_free_¶	0.198		
Stereochemical parameters			
Restraints (r.m.s.d. observed)			
Bond angles (°)	1.86		
Bond lengths (Å)	0.017		
Average isotropic *B* value (Å^2^)	27.1		
ESU†† based on *R*_free_ (Å)	0.061		
Protein residues/atoms	297/2441		
Waters/solvent molecules/ions	431/18/4		

† *R*_merge_ = 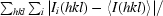 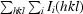, where *I*_*i*_(*hkl*) is the scaled intensity of the *i*th measurement and 〈*I*(*hkl*)〉 is the mean intensity for that reflection.

‡ *R*_meas_ is the redundancy-independent *R*_merge_ (Diederichs & Karplus, 1997 ▶; Weiss, 2001 ▶).

§ *R*_cryst_ = 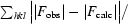 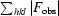, where *F*_calc_ and *F*_obs_ are the calculated and observed structure-factor amplitudes, respectively.

¶ *R*_free_ is the same as *R*_cryst_ but for 5.1% of the total reflections chosen at random and omitted from refinement.

†† Estimated overall coordinate error (Collaborative Computational Project, Number 4, 1994 ▶; Cruickshank, 1999 ▶).

**Table 2:** Summary of crystal parameters and data-collection statistics for Q9HJ63_THEAC in the *C*2 crystal form Values in parentheses are for the highest resolution shell.

	λ_1_ MADSe	λ_2_ MADSe	λ_3_ MADSe
Space group	*C*2		
Unit-cell parameters (Å, °)	*a* = 108.68, *b* = 52.63, *c* = 88.83, β = 121.3		
Data collection			
Wavelength (Å)	0.91837	0.97180	0.97903
Resolution range (Å)	29.67–2.00 (2.05–2.00)	29.66–2.00 (2.05–2.00)	29.66–2.00 (2.05–2.00)
No. of observations	78977	78479	78656
No. of unique reflections	28904	28864	28891
Completeness (%)	99.1 (98.9)	99.0 (96.7)	99.0 (97.3)
Mean *I*/σ(*I*)	9.4 (2.3)	8.5 (2.1)	8.6 (2.0)
*R*_merge_ on *I*† (%)	8.1 (50.0)	9.2 (53.6)	9.6 (58.1)
*R*_meas_ on *I*‡ (%)	10.1 (62.3)	11.4 (66.9)	12.0 (72.5)

† *R*_merge_ = 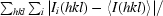 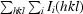, where *I*_*i*_(*hkl*) is the scaled intensity of the *i*th measurement and 〈*I*(*hkl*)〉 is the mean intensity for that reflection.

‡ *R*_meas_ is the redundancy-independent *R*_merge_ (Diederichs & Karplus, 1997 ▶; Weiss, 2001 ▶).

**Table 3:** Summary of crystal parameters, data-collection and refinement statistics for Q9HJ63_THEAC (PDB entry 2gvi) Values in parentheses are for the highest resolution shell.

	λ_1_
Space group	*I*222
Unit-cell parameters (Å)	*a* = 78.60, *b* = 97.65, *c* = 75.27
Data collection	
Wavelength (Å)	1.000
Resolution range (Å)	30.08–1.87 (1.94–1.87)
No. of observations	92701
No. of unique reflections	24247
Completeness (%)	99.8 (99.9)
Mean *I*/σ(*I*)	10.5 (1.7)
*R*_merge_ on *I*† (%)	10.3 (83.1)
*R*_meas_ on *I*‡ (%)	12.0 (96.8)
Model and refinement statistics	
Resolution range (Å)	30.1–1.87
No. of reflections (total)	24246
No. of reflections (test)	1229
Completeness (%)	99.7
Cutoff criterion	|*F*| > 0
*R*_cryst_[Table-fn tfn10]	0.190
*R*_free_[Table-fn tfn11]	0.217
Stereochemical parameters	
Restraints (r.m.s.d. observed)	
Bond angles (°)	1.64
Bond lengths (Å)	0.014
Average isotropic *B* value (Å^2^)	31.1
ESU[Table-fn tfn12] based on *R*_free_ (Å)	0.12
Protein residues/atoms	201/1599
Waters/solvent molecules/ions	129/15/5

† *R*_merge_ = 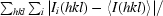 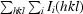, where *I*_*i*_(*hkl*) is the scaled intensity of the *i*th measurement and 〈*I*(*hkl*)〉 is the mean intensity for that reflection.

‡ *R*_meas_ is the redundancy-independent *R*_merge_ (Diederichs & Karplus, 1997 ▶; Weiss, 2001 ▶).

§ *R*_cryst_ = 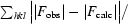 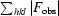, where *F*_calc_ and *F*_obs_ are the calculated and observed structure-factor amplitudes, respectively.

¶ *R*_free_ is the same as *R*_cryst_ but for 5.1% of the total reflections chosen at random and omitted from refinement.

†† Estimated overall coordinate error (Collaborative Computational Project, Number 4, 1994 ▶; Cruickshank, 1999 ▶).

**Table 4:** Summary of crystal parameters, data-collection and refinement statistics for Q2LQ23_SYNAS (PDB code 3d00) Values in parentheses are for the highest resolution shell.

	λ_1_ MADSe	λ_2_ MADSe
Space group	*P*4_1_2_1_2	
Unit-cell parameters (Å)	*a* = 54.36, *b* = 54.36, *c* = 136.72	
Data collection		
Wavelength (Å)	0.9184	0.9782
Resolution range (Å)	29.4–1.90 (1.95–1.90)	29.4–1.90 (1.95–1.90)
No. of observations	118636	118329
No. of unique reflections	16954	16972
Completeness (%)	99.9 (99.9)	99.9 (99.9)
Mean *I*/σ(*I*)	15.6 (2.0)	16.2 (2.0)
*R*_merge_ on *I*† (%)	7.8 (113.4)	7.7 (106.1)
*R*_meas_ on *I*‡ (%)	8.4 (122.3)	8.4 (114.5)
Model and refinement statistics		
Resolution range (Å)	29.4–1.90	
No. of reflections (total)	16902	
No. of reflections (test)	855	
Completeness (%)	99.9	
Data set used in refinement	λ_1 MADSe_	
Cutoff criterion	|*F*| > 0	
*R*_cryst_§	0.233	
*R*_free_¶	0.268	
Stereochemical parameters		
Restraints (r.m.s.d. observed)		
Bond angles (°)	1.60	
Bond lengths (Å)	0.019	
Average isotropic *B* value (Å^2^)	35.2	
ESU†† based on *R*_free_ (Å)	0.16	
Protein residues/atoms	184/1408	
Waters/ions	42/2	

† *R*_merge_ = 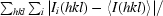 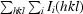, where *I*_*i*_(*hkl*) is the scaled intensity of the *i*th measurement and 〈*I*(*hkl*)〉 is the mean intensity for that reflection.

‡ *R*_meas_ is the redundancy-independent *R*_merge_ (Diederichs & Karplus, 1997 ▶; Weiss, 2001 ▶).

§ *R*_cryst_ = 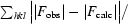 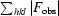, where *F*_calc_ and *F*_obs_ are the calculated and observed structure-factor amplitudes, respectively.

¶ *R*_free_ is the same as *R*_cryst_ but for 5.1% of the total reflections chosen at random and omitted from refinement.

†† Estimated overall coordinate error (Collaborative Computational Project, Number 4, 1994 ▶; Cruickshank, 1999 ▶).

**Table 5:** Metal-ion ligands and coordination geometry in the B8FYU2_DESHY, Q9HJ63_THEAC and Q2LQ23_SYNAS structures

Protein (UniProt designation)	Metal ion	Ligands	Interatomic distance (Å)	Coordination geometry
B8FYU2_DESHY	Ni	His15 NE2	2.2	Tetrahedral
		His17 NE2	1.9	
		Cys19 SG	2.6	
		Cys55 SG	2.2	
	Zn	His15 NE2	2.1	
		His17 NE2	1.9	
		Cys19 SG	2.4	
		Cys55 SG	2.4	
Q9HJ63_THEAC	Zn, N-terminal domain	His16 NE2	2.0	Tetrahedral
		His18 NE2	2.0	
		Cys20 SG	2.4	
		Cys61 SG	2.8	
	Zn, C-terminal domain	Cys174	2.3	
		Cys177	2.3	
		Cys195	2.3	
		Asp198	2.0	
Q2LQ23_SYNAS	Zn, C-terminal doman	Cys165	2.4	Tetrahedral
		Cys168	2.5	
		Cys180	2.4	
		Cys183	2.5	
